# Improvement of Raw and Pasteurized Milk Quality through the Use of Lactoperoxidase Systems (LPSs) along the Dairy Value Chain, under Real Conditions in Ethiopia

**DOI:** 10.3390/foods13081272

**Published:** 2024-04-21

**Authors:** Tigist Ashenafi, Haftom Zebib, Ashagrie Zewdu Woldegiorgis

**Affiliations:** 1 Center for Food Science and Nutrition, College of Natural Sciences, Addis Ababa University, Addis Ababa P.O. Box 1176, Ethiopia; tigistashenafich@gmail.com (T.A.); hzhzebib6@gmail.com (H.Z.); 2 Tigray Agricultural Research Institute-Livestock and Fishery Core Process, Mekelle P.O. Box 492, Ethiopia

**Keywords:** LPS, total bacterial count, raw milk, pasteurized milk, *E. coli*

## Abstract

Lactoperoxidase systems (LPSs) can enhance the microbiological quality of raw milk when there is lack of cooling facilities. In this study, a total of 250 milk samples were collected from farmers, collectors, and factories. Experimental samples were both LPS-activated morning and overnight milk. The samples were tested with several chemical and microbiological tests, such as total bacterial count (TBC), total coliform count (TCC), and *Escherichia coli* count (EC). Results indicated that all LPS-activated milk samples had a higher quality than all the control samples. For instance, both the morning and overnight farm milk samples had mean TBCs of 5.79 log and 6.55 log cfu/mL, which is significantly (*p* < 0.05) lower than the control samples’ mean TBC of 6.73 log and 7.31 log cfu/mL, respectively. When this was compared with the Ethiopian Standard, 51.4% of morning and 39.5% of overnight farm milk with LPS activation met the acceptable quality, while only 28% of morning and 15.7% of overnight control milk met the standard. Moreover, LPS activation has also significantly improved the shelf life of collectors’ raw milk and pasteurized milk at the factories. Therefore, a better hygienic practice with LPS application can be practiced in conditions that lack cooling infrastructure and electricity.

## 1. Introduction

Milk contains important nutrients and satisfies people’s nutritional needs. However, milk is an ideal medium for the growth and multiplication of diverse microorganisms, resulting in its early deterioration [1]. Contaminating bacteria may multiply rapidly and render it unsuitable for processing and/or unfit for human consumption. The most commonly used preservation technology to stop or retard the deterioration of milk and to reduce the postharvest loss of milk from farmers to collection centers is cooling facilities. However, the lack of available capital, lack of electricity, less-developed road systems, high operational costs, frequent equipment breakdowns, a lack of spare parts, and difficulties in equipment repair in rural areas are major challenges to milk collection centers [2]. There is also the informal addition by the farmers of formalin to raw milk for preservation, to increase the shelf life for the long-distance transportation to milk collection centers in Ethiopia [3]. Formalin is highly toxic, causes liver and kidney damage, and is considered carcinogenic. To tackle this problem, the use of LPSs in areas where there is currently no adequate infrastructure for the collection of raw milk has been developed and applied [4,5].

Lactoperoxidase (LPO) is an enzyme naturally found in milk. One of its unique biological functions is its antibacterial effect in the presence of hydrogen peroxide (H_2_O_2_) and thiocyanate. Both of these substances are naturally present in milk, in varying concentrations. The natural bacteriostatic effect of LPS lasts for at least one hour after milking [6]. For a continued effect, the system has to be activated. This activation can be achieved by adding about 10 parts per million (ppm) (5 ppm is naturally present) of thiocyanate (preferably in powder form) to the raw milk, to increase the overall level to 15 ppm [7].

Lactoperoxidase (LPS) is effective against most microorganisms, with the activation of thiocyanate and hydrogen peroxide to oxidize and produce the antimicrobial agent hypothiocyanite (OSCN), which exerts its action by oxidizing the sulfhydryl groups of proteins to disulfides. Thus, LPSs enhance the shelf life of raw milk from 7 to 26 h, at different storage temperatures (15 to 30 °C) [4]. LPSs also have a bactericidal effect on most milk-borne pathogenic bacteria (*Escherichia coli*, *Salmonella* spp., *Campylobacter* spp., *Staphylococcus aureus*, *Listeria monocytogenes*, *Yersinia enterocolitica*, and *Brucella melitensis*). However, the effectiveness of LPSs depends on the raw milk’s initial microbial load on the fluid milk’s quality and shelf life [8]. The spoilage microflora in pasteurized milk is completely different from that found in raw milk, which consists mainly of post-pasteurization contaminants [9]. There is also an incidence of pathogens in pasteurized milk, and foodborne outbreaks, due to inadequate pasteurization or post-pasteurization contamination, have been reported in Ethiopia. Residual lactoperoxidase activity plays a role in the quality of pasteurized milk and dairy products, in general [8].

Ideally, milk should be cooled to <6 °C immediately after milking (2–3 h) and should be transported to dairy plants as soon as possible, under the cold chain. However, in some countries, including Ethiopia, the establishment of cooling units is impractical because of a lack of capital, a lack of electricity, insufficient transportation systems, and high operational costs. Insufficient cold storage systems eventually lead to the excessive multiplication of bacteria and increase the acidity of raw milk far beyond the level acceptable for processing [10]. It is, therefore, important to look for alternative methods for retarding bacterial growth in raw milk during collection and transportation to the dairy processing plant.

The production of high-quality milk should be a priority for good-quality end products with a long shelf life and for marketing value-added products [11]. But it is difficult to achieve this, due to a lack of cooling facilities, high ambient temperatures, and insufficient infrastructure for milk transportation to the market [12]. The fact that the few dairy enterprises currently operating in and around Addis Abeba are operating below capacity, due to a lack of milk supply, does not imply a gap between milk production and demand, as only 5% of the milk produced in rural areas is marketed [13].

Much work has been conducted on both the LPS in raw cow milk and the activation of the system prior to heat treatment, in different countries under laboratory conditions [14,15]. So far, in Ethiopia, few papers have been published on the evaluation of LPSs during raw milk storage periods at different temperatures, under laboratory conditions at a controlled temperature [16,17]. These studies did not address the quality and shelf life of milk during its transportation to processing plants under real-world conditions. In addition to this limitation, there is no information on the effect of LPSs on pasteurization efficiency or residual LPSs on the keeping of pasteurized milk in the context of poor handling, unhygienic practices, or where there is a high microbial load in raw milk. Therefore, the objectives of this research were (i) to evaluate the effect of LPSs on the quality of raw milk during storage and transportation at the farm under real conditions; (ii) to evaluate the effect of LPSs during storage and/or transportation to collection centers under real conditions prior to processing; (iii) to evaluate the effect of LPSs on the quality and shelf life of pasteurized milk during post-pasteurization storage; and (iv) to compare LPS-activated milk quality with Ethiopian standards.

## 2. Materials and Methods

### 2.1. Study Area

The study districts for sample collection were selected according to the Central Statistical Agency, based on milk production potential. The study districts include urban and peri-urban areas of the Oromia region (Selale, Holeta, Bishoftu/Debrezeyit, and Asella) [18]. In each study district, smallholder dairy farmers, collectors, and milk factories were included.

### 2.2. Study Design, Sample Size, and Sampling Technique

A cross-sectional study was used to collect primary samples. Dairy farmers were selected using a simple random technique, whereas collectors and factories were selected purposefully. In this study, dairy farmers (n = 40), collectors (n = 4), and processors (n = 4) participated. Table 1 shows the sample size for treatments along the value chain at each study site.

The sampling of raw milk from farmers, collectors, and factories was conducted according to ES ISO [19]. Milk in the farmers’ buckets (small vessels) was thoroughly mixed using a suitable dipper. Then, about 500 mL of sample was taken from each farmer’s bucket. From this, about 250 mL of milk sample was used for LPS activation and control. At the collection centers, five different aluminum or plastic cans were randomly selected from the collected milk. Then, each container was thoroughly mixed using stainless steel dippers. From this, about 250 mL of milk sample was used for LPS treatment and control. After milk was received by milk factories, composite samples (3 L) were sampled from different milk tankers for each treatment analysis and pasteurized milk shelf-life evaluation.

### 2.3. Collection of Laboratory Sample, Transportation, and Storage

All samples were collected from January 2022 to October 2022. Raw milk samples were collected from farmers, collectors, and milk factories (prior to pasteurization). Laboratory samples for microbiological and physicochemical examinations were taken separately, using sterilized equipment, sampling apparatus, and containers, in accordance with ES ISO [19]. Those samples for microbiological analysis were taken first. Raw milk samples were collected in sterile, screw-capped, clean plastic bottles (250 mL capacity). Proper labeling was provided for each sample. Morning samples were transported under real conditions, whereas overnight samples were transported in an ice box at 2–8 °C, to the Addis Ababa University Center for Food Science and Nutrition laboratory. Samples were analyzed immediately for microbiological determination. Those samples for chemical quality analysis were stored at 2–8 °C.

### 2.4. Activation of LPSs in Raw Milk

LPS activation in raw milk was performed according to Codex Alimentarius [4]. The LPS was activated by the addition of 14 mL of freshly made sodium thiocyanate (1 mg/mL solution) (General Chemical Division, New York, NY, USA) per liter of milk, in order to provide a source of the thiocyanate (SCN-) ion. In total, 10 mL of freshly made, 1 mg/mL hydrogen peroxide solution (BDH Chemicals Ltd., Poole, England) was added to the milk after the mixture had been completely stirred for 1 min.

### 2.5. Experimental Trial and Procedure

The raw milk experimental samples from farmers, collectors, and milk factories (prior to pasteurization) were grouped into two treatments, as follows: LPS-activated morning milk and LPS-activated overnight milk. The control consisted of LPS-untreated morning and overnight milk samples in each value chain. Overnight milk samples from each value chain were activated with LPS and were kept overnight (12 h) at farmers, collectors, and milk factories under real conditions; then, laboratory samples were transported using a portable refrigerator (Dometic, CFX-50 W, Shenzhen, China) to the laboratory, whereas morning milk samples were activated with LPS and then directly transported under real conditions to the laboratory. For pasteurization purposes, the LPS was activated overnight and the morning milk collected from milk factories was pasteurized and evaluated for shelf life. All analyses were run in duplicate.

### 2.6. Validation of Pasteurization and Shelf Life Evaluation of the Pasteurized Milk

The validation of the pasteurization method can be accomplished in two ways. Firstly, by validating the process equipment temperature (72 °C) and holding time (30 min) and, secondly, by validating the product via pathogen reduction and growth. If available, the effectiveness of the pasteurization treatment can also be validated by verifying the presence of the phosphatase level in the pre- and post-pasteurization milk. Pasteurization systems are designed to provide a 5 log reduction in the microbial load. With pasteurization, not only are pathogenic microorganisms killed, but also a wide range of spoilage organisms are destroyed [10]. Based on the above criteria, both process equipment and product validation were performed prior to the study by measuring the temperature with an electronic thermometer to determine whether the water bath display reading was correct or not; product validation was also performed by detecting the TBC and TCC of the samples both before and after pasteurization, to determine the effectiveness of the pasteurization.

The pasteurization of the samples was carried out at a laboratory scale using a water bath, as described by Tetrapack [20]. All plastic containers containing samples (LPS-activated and control morning and overnight milk) were immersed in a water bath (Biobase, WB-82, Jinan, China) and pasteurized at 65 °C for about 30 min. Then, the samples were cooled in running water and were kept in the refrigerator at 2–8 °C for 10 days, to mimic the real storage condition. Afterwards, samples were drawn at 0, 2, 4, 6, 8, and 10 days of storage to evaluate keeping quality and shelf life.

### 2.7. Assessment of Microbiological Quality of Milk

#### 2.7.1. Total Bacterial Count (TBC)

The presence of mesophilic bacteria in mL of diluted milk samples was tested, according to the FDA [21], using the pour plate method with plate count agar. Milk samples were homogenized and serially diluted by adding 1 mL of the sample into 9 mL of 0.1% peptone water for initial dilution and by transferring 1 mL of the previous dilution into 9 mL of peptone water. Appropriate dilutions were placed on Petri dishes and pour-plated with 10 to 15 mL of molten plate count agar for TPC. The sample and agar were gently mixed and permitted to harden on the bench for roughly 30 min, using alternating clockwise and anti-clockwise rotations. The plates were inverted and incubated at 32 +/−°C for 48 h. Plates inoculated with a sample dilution yielding between 25 and 250 colonies were counted after incubation. The number of bacteria in a milliliter of milk was determined using the FDA (2000) method, after colony counts were performed with a colony counter.

(1) $$N=\frac{\sum C}{V\;(n+n_{2\left(0.1\right)d})}$$

where C = the sum of colonies on all plates counted

V =the volume applied to each plate
N = the number of plates counted at first dilution
n_2_ = the number of plates counted at second dilution
d = dilution from which first count was obtained
N = the average plate count.

#### 2.7.2. Enumeration of Total Coliform Count (TCC) and *E. coil*

The protocols for counting total coliforms and *E. coli* counts in milk samples were followed, according to 3M Food Safety [22]. One milliliter of raw and pasteurized milk was serially diluted in nine milliliters of 0.1% peptone, for up to five and three dilutions, respectively. Prior to applying the 1 mL diluent to the *E. coli*/coliform count plate Petri film for quantification of *coliforms* and *E. coli*, the diluent was vortexed to homogenize the serial dilution. One mL of diluent was taken from the supernatant for serial dilution. Then, the top film was lifted and 1 mL of sample suspension was dispensed onto the inoculation area. The Petri film plates were incubated in a horizontal position at 35 ± 2 °C for 24 ± 2 h. The plates were incubated for an additional 24 ± 2 h (48 ± 4 h total) until colonies of sufficient size to count were observed. The total coliform count consisted of both red and blue colonies associated with gas; blue colonies with a gas bubble were counted as *E. coli* [22].

(2) N=∑C×V×d

where ∑C = the sum of the colonies counted on the two Petri films

V = the volume of culture plates
d = the dilution corresponding to the first dilution retained

### 2.8. Determination of Milk Quality Indicators

#### 2.8.1. Determination of pH

The pH of the samples was measured using a digital pH meter (HFCC, PHS-3DW, Hong Kong, China), according to the manufacturer’s manual. Before analyzing the sample, the pH meter was calibrated using 4.0 and 7.0 buffer solutions.

#### 2.8.2. Determination of Titratable Acidity (TTA)

The TTA of the milk samples was determined, according to AOAC [23], by measuring 10 mL and adding water (40 °C) to a volume of 105 mL. After vigorous shaking, the mixture was filtered and an aliquot of the filtrate (25 mL) was titrated with 0.1 N sodium hydroxide solution, using phenolphthalein as an indicator. The TTA was expressed as % lactic acid and calculated using the formula below.

(3) $$Lactic\;acid\;\left(\%\right)=\frac{Volume\;of\;NaOH\;used\times 0.009}{Volume\;of\;milk\;sample\;used}\times 100$$

#### 2.8.3. Determination of Thiocyanate Concentration

This was measured using trichloroacetic acid (TCA) as the ferric complex, at 460 nm absorbance [4]. Milk samples (4.0 mL) were mixed with 2.0 mL of a 20% TCA solution. The mixture was blended well and then allowed to stand for at least 30 min. It was, thereafter, filtered through a suitable filter paper (Whatman No. 40). The clear filtrate (1.5 mL) was then mixed with 1.5 mL of the ferric nitrate reagent (16.0 g Fe (NO_3_)). A total of 3.9 mL H_2_O was dissolved in 50 mL of 2M HNO_3_ and was then diluted with distilled water to 100 mL; the absorbance was measured at 460 nm using a UV spectrophotometer. As a blank, a mixture of 1.5 mL of ferric nitrate solution and 1.5 mL of water was used. The measurement was carried out within 10 min of the addition of the ferric nitrate solution, as the colored complex is not stable for any length of time. The concentration of thiocyanate was then determined by comparison with standard solutions of known thiocyanate concentrations (10, 15, 20, and 30 g/mL of thiocyanate).

### 2.9. Statistical Analysis

The obtained data were analyzed using STATA Version 20 and XLSTAT for Microsoft Excel Version 2015. 2.01. Independent t-tests were used to compare the quality of LPS-activated liquid milk and the control. One-way ANOVA and descriptive statistics were used to compare the mean results. DMRT was used to test between mean pairs and was accepted at a probability of 0.05.

## 3. Results

### 3.1. Effect of LPSs on the Microbiological Quality of Raw Milk along the Dairy Value Chain

The effects of LPSs on the microbiological quality of raw milk collected from dairy farmers are presented in Table 2. Results indicate that there is a significant difference (*p* < 0.05) in the TBC between the different treatments in both the morning and overnight milk samples collected from farmers. In the morning milk samples, the mean TBC of the LPS-activated samples was 5.79 log cfu/mL, which was significantly lower than the control samples, which had a mean TBC of 6.73 log cfu/mL. This indicates a decrease in TBC of 0.94 log cfu/mL in the LPS-activated samples, as compared to the control samples. Similarly, in the overnight milk samples, the mean TBC of the LPS-activated samples was 6.55 log cfu/mL, which was significantly lower than the TBC of the control-treated samples, which had a mean TBC of 7.31 log cfu/mL. This indicates a decrease in TBC of 0.76 log cfu/mL in the LPS-activated samples, as compared to the control samples.

In the present work, the use of an LPS indicates a significant reduction in the TCC of farmer milk. The mean TCC values of morning and overnight milk with LPS activation were 3.77 log cfu/mL and 4.19 log cfu/mL, respectively. In comparison, the mean TCC values of the control samples were 4.7 log cfu/mL for morning milk and 5.04 log cfu/mL for overnight milk. This result suggests a reduction in TCC of 0.9 log units in morning milk and 0.86 log units in overnight milk, when LPS activation was used.

The activation of LPSs on morning and overnight milk significantly (*p* < 0.05) reduced the growth of *E. coli*, as compared to that of activated control morning and overnight milk samples, produced, stored, and transported under real conditions. The mean *E. coli* count in both control morning and overnight milk samples was 0.84 and 0.52 log cfu/mL, respectively (Table 2). The mean *E*-*coli* count in LPS-activated milk was lowered by 0.66 and 0.52 log cfu/mL, as compared to control morning and overnight milk samples, respectively.

Table 2 summarizes the microbiological quality of collectors’ milk samples with LPS activation and control samples. The presence of TBC was significantly (*p* < 0.05) higher in the control samples compared to the LPS-activated samples. The mean TBC of morning milk with LPS activation was 6.15 log cfu/mL, while the mean TBC of control morning milk was 6.99 log cfu/mL. Similarly, the mean TBC of overnight milk with LPS activation was 6.96 log cfu/mL, whereas the mean TBC of control overnight milk was 7.78 log cfu/mL.

There was a significant (*p* < 0.05) difference in the TCC between the activated samples and control samples under the same conditions. In the study, a reduction of 1 log unit in TCC was observed in the LPS-activated morning milk samples, compared to the control. Similarly, a reduction of 1.1 log units in TCC was observed in the LPS-activated overnight milk samples, compared to the control samples. The mean TCC values reported in the study were 4.63 log cfu/mL for the activated morning milk sample, 5.27 log cfu/mL for the activated overnight milk sample, 5.63 log cfu/mL for the control morning milk sample, and 6.41 log cfu/mL for the control overnight milk sample.

Both morning and overnight milk samples with LPS activators had significantly (*p* < 0.05) lower *E. coli* counts than the control samples. The mean *E. coli* count of morning milk with LPS activation was 0.2 log cfu/mL, while *E. coli* was not detected in activated overnight milk. *E. coli* was found in the control morning and overnight milk samples, with concentrations of 0.93 log cfu/mL and 0.63 log cfu/mL, respectively.

### 3.2. Effect of LPSs on the Microbiological Shelf Life of Pasteurized Milk

#### Effect of LPSs on the TBC and TCC of Pasteurized Milk

LPS-activated and control milk collected during overnight storage had no significant difference (*p* > 0.05) in TBC until the second day of storage; however, on the fourth day of storage, control overnight milk showed a significant increase in TBC, as compared to the LPS-activated samples, and, on the sixth day of storage, the control sample failed to meet the EASs (East African Standards) [24], while activated samples were acceptable until the tenth day of storage. Similarly, the TBC of control morning pasteurized milk failed to meet the EASs (not exceeding 30,000 cfu/mL or 4.47 log cfu/mL) (EAS, 2006) on the eighth day of storage, while LPS-activated morning pasteurized milk reported 3.9 log cfu/mL on the tenth day of storage, which was below the EASs [24].

**Figure 1:**
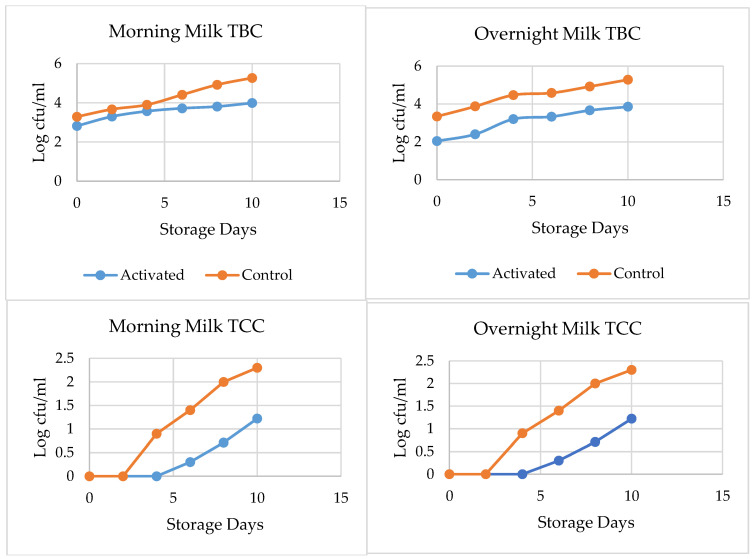
Effect of LPS activation on TBC (**top**) and TCC (**bottom**) of pasteurized milk, throughout 10 days of storage.

The effect of LPSs on the TCC of overnight and morning milk is shown in Figure 1. LPS activation, prior to processing overnight milk during storage, inhibits TCC growth significantly, as compared to the control sample during the tenth day of storage. LPS-activated overnight milk had a 0.7 log unit TCC, while that of the control had 2.5 log CFU/mL, on the eighth day of storage. The LPS-activated morning milk indicated only a 0.5 log unit growth in TCC from day 0 to the eighth day of storage, while a 1.9 log unit growth was observed in the activated control sample.

According to the Ethiopian Standards Agency (ESA) [25], it is recommended for pasteurized milk to contain no more than 10 cfu/mL or 1 log cfu/mL TCC, as shown in Figure 1; the TCC of LPS-activated morning milk met the standard up to the tenth day of storage, while that of the control-activated morning milk failed to meet the standard on the sixth day of storage. In the case of overnight milk, the control-activated samples stored overnight for 12 h under real conditions before processing did not meet the ESA’s 2009 recommendations on the fourth day of storage, while the LPS-activated samples met the standard until the tenth day of storage. This indicates that LPS activation before processing can improve the quality of pasteurized milk until the tenth day of storage.

### 3.3. Comparison of Microbiological Quality with Ethiopian Standards

A comparison of microbiological quality of LPS-activated and control samples with the ESA is presented in Table 3. The results show that 51% of LPS-activated morning samples and 40% of LPS-activated overnight samples collected from farmers met the ESAs requirements for TBC, respectively. Both activated samples had a higher percentage of passes than their control samples. As concerns samples from collectors, only 45% of morning and 15% of overnight samples met the minimum requirements set by the ESA [25]—a limit of no more than 50,000 cfu/mL.

Of the samples from farmers that were analyzed, the TCC values for LPS-activated morning samples (82%) and control samples (51%) were found to fulfil the minimum requirements of the ESA, while LPS-activated overnight samples (60.55%) had also passed the minimum requirements. The results also show that, with the use of LPS activation in collectors’ milk, 70% of the morning milk samples and 30% of the overnight milk samples passed the ESA’s [25] limit of no more than 1,000,000 cfu/mL.

According to the study’s *E. coli* count from farmers’ milk, 23.6% of morning control samples, 25.6% of overnight control samples, as well as 10.3% of LPS-activated morning milk, did not meet the ESA safety requirements to be nil in marketable dairy products. From collectors’ milk, 90% of morning activated milk, 65% of morning control milk, and 75% of overnight control milk passed the ESA’s minimum requirements. Results also indicate that *E. coli* was not detected in overnight milk that was activated with the LPS from either farmers’ or collectors’ milk.

### 3.4. Effect of LPSs on Quality Test of Raw and Pasteurized Milk along the Dairy Value Chain

#### pH, TTA, and Thiocyanate Concentration

The effect of the LPS on pH, TTA, and thiocyanate concentrations in raw milk samples is presented in Table 4. There were significant (*p* < 0.05) differences in pH values between the LPS-activated and control samples collected from farmers. The pH values of the control morning (6.33) and overnight sample (6.04) were much lower than those of the LPS-activated morning milk (6.51) and overnight samples (6.31), though the pH values of all samples were lower than the normal milk pH value (6.6–6.7).

We also found a significant (*p* < 0.05) difference in TTA values between the control and activated morning samples. However, statistical analysis did not show a significant (*p* > 0.05) difference between activated and control morning samples.

In this work, there was no significant difference (*p* < 0.05) between the average thiocyanate content of morning and overnight milk with LPS activation, compared to the control. The highest thiocyanate concentration recorded was 18.3 ppm, while the lowest was 8.92 ppm. The mean thiocyanate content of morning and overnight milk with LPS activation observed in this study was 13.64 ppm and 15.14 ppm, respectively.

The effects of LPSs on pH, TTA, and thiocyanate concentration of collectors’ milk are show in Table 4. In this work, the mean TTA of LPS-activated morning and overnight milk samples was 0.23 and 0.27, while the mean TTA of control morning and overnight milk samples was 0.26 and 0.35, respectively, which were higher values than the ESA (0.1 to 0.17%). The mean pH values of LPS-activated morning (6.4) and overnight collectors’ milk (6.3) were significantly higher than those of control morning (6.22) and overnight (6.06) milk samples.

In the present work, the thiocyanate ion (SCN-) content of raw milk collected from collectors was lower in the control samples than in the LPS-activated samples. There were no statistically significant differences (*p* > 0.05) in all study samples. The thiocyanate levels in the LPS-activated samples increased by 0.08 ppm in the morning and 1.03 ppm in the overnight milk, compared to the control samples.

**Figure 2:**
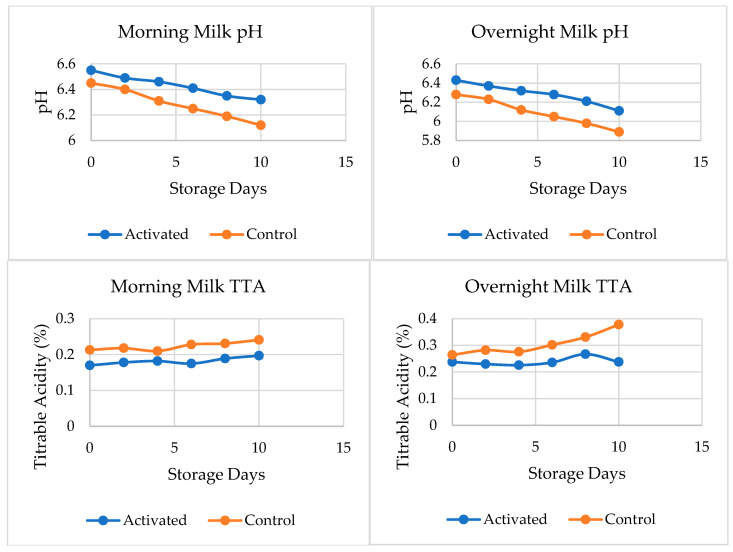
Effect of LPS activation on pH (**top**) and titratable acidity (**bottom**) of pasteurized milk, throughout 10 days of storage.

The effect of LPS activation on the pH and TTA of pasteurized morning and overnight milk during storage is depicted in Figure 2. The pH of both types of morning milk samples did not differ significantly (*p* < 0.05) until the 4th day of storage, after which control milk showed a significant drop in pH compared to LPS-activated milk (Figure 2). On the other hand, the TTA of both types of morning milk samples differed significantly (*p* < 0.05) starting from the 0th day of storage until the 10th day. The TTA of pasteurized morning milk control samples was significantly (*p* < 0.05) higher than that of LPS-activated milk.

## 4. Discussion

### 4.1. Effect of LPSs on the Microbiological Quality of Farmer’s Milk

In the present work, the microbiological quality of raw milk samples varied when treated with LPSs. The TBC of raw milk from farmers varied between LPS-activated morning milk and activated overnight milk samples, when compared to control. These reductions suggest that the use of the LPS had a positive effect on retarding and multiplying bacterial loads. The present results were consistent with the findings of Sepulveda and Munoz [26], who discovered significant differences in the bacterial count between samples of milk with and without LPS activation, after 12 h of storage at an ambient temperature. The study by Ponce [27] also demonstrated that activation of the LPS had an effect on viable mesophiles, due to the bacteriostatic effect with, at first, a light reduction in bacterial load and an increase in its bactericidal potential after 4 h of activator inoculation, reaching its maximum expression at 9 h and, later, decreasing upon reaching 12 h. The study by Amenu et al. [28] found a 1.07 log cycle TBC reduction after 6 h of storage at 30 °C in raw milk. Similarly, Nigussie and Seifu [29] reported an 8.57 log cfu/mL TBC for samples without LPS activation, after 7 h of storage at 22–24 °C; whereas, samples with LPS activation, under the same conditions, had a 7.5 log cfu/mL TBC.

In the present research, the TCC in morning LPS-activated milk samples was lower than in the control. This indicates that the LPS exhibited a bacteriostatic effect against TCC in milk under real milk production, storage, and transportation conditions. The present result is in agreement with [30], who reported a 1.28 log unit TCC reduction at 7 h of storage at 30 °C and a 1.73 log cfu/mL reduction was reported by Nigussie and Seifu [29] at 7 h of storage at 22–23 °C. The effectiveness of the LPS for raw milk preservation on TCC can be influenced by initial milk quality and the circumstances of the experiment [30,31].

LPS activation in morning and overnight milk samples reduced the growth of *E. coli*, as compared to that of the control. This implies that the LPS had a significant effect on reducing the *E. coli* count. A study by Thomas and Aune [32] stated that LPSs inhibited succinate-dependent respiration in *E. coli*, which correlated with the loss of bacterial viability. A study in Ethiopia by Amenu et al. [28] demonstrated a significant reduction in the growth of *E. coli*, as compared to control samples, at 6 h of storage. *E. coli* have a high metabolic activity and, thus, the oxidation product of the LPS may not have been able to counteract *E. coli* multiplication at ambient temperature [33], which may have been the cause of LPS failure, resulting in the presence of bactericidal effects in morning milk. The research conducted by Pruitt and Njage [34] also reported that the variability of the bactericidal properties of milk can be caused by variations in the quantities of peroxidases contained in different milk samples.

### 4.2. Effect of LPSs on the Microbiological Quality of Collector’s Milk

In this study, the presence of TBC was higher in the control samples than in the LPS-activated samples (Table 2). This implies that LPS activation had a positive effect on the retardation of bacterial multiplication, as compared to the control groups. However, a relatively higher initial bacterial load, which was beyond the recommended standard, was observed in both LPS-activated and control samples. This could be due to the large-volume mixing of different initial densities of bacteria in raw milk from different farmers, through formal and informal means, at the milk collectors, thereby elevating the load of bacteria in the mixed raw milk.

The higher TBC could be influenced by a lack of knowledge about clean milk production, milk contamination from the hands of handlers, the use of plastic containers for collecting and keeping milk, further contamination of the milk during transportation, and the absence of cooling systems at milk selling points. Poor quality can be attributed to the milkmen’s lack of cleanliness, the insecurity of the water used for cleaning purposes, the udder of the cow, the milking environment, and the milking equipment, which could be the primary sources of the initial milk contamination [35].

In the present investigation, a reduction in the TCC of the activated samples was observed, as compared to the control samples under the same real conditions. This reduction suggests that LPS activation can maintain the initial quality of raw milk by inhibiting the growth and multiplication of TCC. The presently found values were harmonious with the results obtained by Amenu et al. [28], who found differences with respect to the coliform count between samples of milk both with and without the activation of the LPS at 7 and 12 h of storage, since the said samples with the activation of the LPS showed a 1.23 and 1 log unite coliform count reduction at 6 and 12 h of storage, respectively.

The current findings are also in agreement with the findings of Nigusse and Seifu [29] (2007), who reported a decrease in TCC in cow milk after 7 h of activation of the LPS, as compared to the control treatment at ambient storage temperatures between 22 and 23 °C. The study by Campos-Vallejo et al. [36] also reported 0.1, 0.56, 1.67, 2.66, and 1.8 log units of total coliform reduction at 0, 3, 6, 9, and 12 h of storage at ambient temperature, respectively. LPS activation affects the TCC of cow milk, confirming its ability to preserve milk quality and the combination of bactericidal and bacteriostatic effects on mesophilic bacteria, including coliforms [37]. According to the FAO/WHO [14], the hygiene of milk plays an important role in extending the shelf life of LPSs [38]. A high microbial load in normal milk demonstrated the contamination of the milk. Likewise, a high load of coliform indicates contamination from manure or soil. Other possible causes of contamination might be the hands and arms of the milking person, the water, and the milking environment [39].

In the present work, activated milk samples collected from collectors had a lower *E. coli* count than samples without LPS activation. This could be due to the bactericidal effect of LPS in overnight milk samples and the inhibitory effect of LPS in morning milk samples. The mean *E.coli* counts of morning and overnight milk samples without LPS activation were 0.93 log cfu/mL and 0.63 log cfu/mL, respectively, whereas, morning milk samples with LPS activation had a 0.2 log cfu/mL. However, *E. coli* was not detected in overnight milk with LPS activation.

Compared to the morning sample without LPS activation, the *E. coli* count was 0.73 log lower with LPS activation. The study by Ozer [10] stated that both Gram-positive and Gram-negative bacteria can be affected by the LP system, either reversibly or irreversibly. The capacity of cells to recover from inhibition depends mainly on environmental conditions (temperature and pH) and on the particular strain. In a study conducted at Haramaya University, Amenu et al. [28] reported that, after 6 h of storage at 30 °C, LPS activation in a camel milk sample with *E. coli* significantly inhibited *E. coli* growth. Since *E. coli* is a mesophilic bacteria that thrives in temperatures between 21 and 49 °C, it is possible that the ambient temperature and contamination during transportation under real conditions can explain why the bactericidal effect of the LPS against *E. coli* was not noticed in morning milk in the current study.

### 4.3. Effect of LPSs on the Microbiological Shelf Life of Pasteurized Milk

In this study, LPS-activated samples had a higher shelf life of pasteurized milk. The TBC and the TCC of LPS-activated samples were lower. This can be explained by the inhibition effect of the combination of LPS activation and a low refrigeration temperature. The prolonged shelf life of LPS-activated pasteurized milk is also explained by the fact that certain bacteria become weakened by the effect of LP treatment, making them more susceptible to heat treatment [40]. The LPS has the greatest impact on psychotropic bacteria and certain heat resistant spore-forming bacteria, which are normally the cause of the spoilage of pasteurized milk under refrigerated storage conditions.

The residual LPS in pasteurized milk in standard pasteurization was sufficient to catalyze the reactions between thiocyanate and hydrogen peroxide [2,14,41]. However, time taken for storage, transportation, and improper field-level practices contributed to the high bacterial density of raw milk, which can impair the microbial quality of pasteurized milk and also the effectiveness of the LPS in pasteurized milk. The LPS has been shown to improve the shelf life of bovine milk, when activation is carried out prior to pasteurization [42].

The LPS can continue to function under normal pasteurization temperature and time [8]. This is because lactoperoxidase is the most heat-stable enzyme in milk and is only inactivated above 78 °C for periods of 15 s. There was a sufficient residual enzyme activity for the LPS to be operational and to exert an effect on the keeping quality of the milk pasteurized at standard pasteurization temperatures. Moreover, LPS-activated milk produces pasteurized milk of better bacteriological and storage quality, due to the enhanced thermal destruction of milk spoilage bacteria [8,43].

### 4.4. Effect of LPSs on the Quality of Farmer’s Milk

In the present work, the pH of the control samples was much lower than that of the LPS-activated samples. This can be explained by the higher pH-maintaining capability of the LPS compared to milk without activation. A study by Fonteh et al. [44] reported the shelf life of LPS-activated milk, which was kept fresh, without a considerable drop in pH, while the LPS-control milk stored at room temperature (21–23 °C) became spoiled only after an additional 3 h of storage.

The TTA results indicate that the speed of acid production was lower in activated overnight milk than in control overnight milk. The decreased acid production rate might be due to inhibitory compounds, such as hypo-thiocyanate, forming during the oxidation of thiocyanate and hydrogen peroxide, during the activation of the LPS in milk [2,45]. The acidity difference observed between the LPS-activated and control samples in the current study was much lower than was observed in the study carried out by Asaah [46]. He found a 29% lower lactic acid content in activated milk than in control milk, after 16 h of activation at ambient temperature, this was also 15% lower in a water bath (20 °C).

The thiocyanate content of the present result is similar to the findings of Fonthe [44], who reported an average value of 13.60 ppm for cow milk in Cameroon. The thiocyanate concentration obtained in this study was higher than that of Nigussie and Seifu’s (2007) findings, who found an average value of 7.38 ppm in Kombolcha, Eastern Ethiopia. The concentration of thiocyanate in milk can be influenced by the animal’s age, health, species, breed, lactation stage, and feed type [14,47]. The range of thiocyanate ion concentrations in milk from individual cows was 0.05–0.62 mmol/L, while the range of concentrations of thiocyanate in bulk milk was 0.1–1.18 mmol/L, with an average of 0.14 mmol/L [27].

### 4.5. Effect of LPSs on the Quality of Collector’s Milk

The mean pH values of the LPS-activated morning and overnight collectors’ milk were significantly higher than those of the control samples. These values imply that the use of LPS activation maintained a stable pH in morning milk. In general, the mean pH values of both the LPS-activated and control samples obtained from the current study were below the recommended range for fresh cows’ milk (6.6–6.7). This might be due to the 12 h storage time between milking and analysis, under real conditions, and the further exposure of milk to high contamination during transportation under real conditions. The pH values of the LPS-activated milk samples dropped slowly, while the control milk showed a faster drop in pH [18].

In this study, we observed a significant increase in acid production in control morning milk samples during transportation, as compared to the LPS-activated milk acidity, which indicates the positive effect of the LPS on retarding the growth of lactic acid bacteria during storage under real conditions. The research conducted by Kumar and Mathur [48] reported an increase in the TTA of milk stored in similar conditions of elevated temperatures.

In this work, the thiocyanate level in both the LPS-activated samples was higher than in the control samples. This result is similar to the finding of Fonthe [44], who reported an average value of 13.60 ppm for cow milk in Cameroon. The thiocyanate concentration obtained in this study was higher than that of Nigusse and Seifu’s [29] study, who found an average value of 7.38 ppm in Kombolcha, Eastern Ethiopia. The concentration of thiocyanate in milk can be influenced by the animal’s age, health, species, breed, lactation stage, and feed type [14,47].

### 4.6. Effect of LPSs on the Keeping Quality of Pasteurized Milk

In this study, the control samples showed a significant drop in pH values compared to the LPS-activated milk samples. This suggests that LPS activation could help to maintain the pH of pasteurized milk during storage, by retarding the multiplication and growth of bacteria. The TTA of pasteurized morning milk control samples was significantly higher than that of LPS-activated milk. This indicates that LPS activation retards acid production in pasteurized milk and, hence, could be useful in extending the shelf life of pasteurized milk.

LPS activation had no significant effect on the pH or TTA of overnight milk. Therefore, the study suggests that LPS-activated milk samples should be processed before storage for 12 h or more. This is because storage time could significantly decrease the effectiveness of the LPS in retarding acid production in pasteurized milk. The results suggest that LPS activation can be useful in extending the shelf life of pasteurized morning milk by maintaining the pH and retarding acid production. However, the effectiveness of the LPS could vary with the type of milk and the storage time.

In Ethiopia, pasteurized milk is predicted to have a shelf life of 5 days when kept in a refrigerator, as well as a pH of 6.6 when freshly processed. According to Ethiopian customers, pasteurized milk should pass the sweet test and be re-heatable, which means that the milk’s pH should be above 6.4 and its lactic acid content should be below 0.22%. Based on this criterion, the results suggest that LPS activation enhanced the shelf life of morning pasteurized milk under refrigerator storage.

In this study, the LPS-activated morning milk remained acceptable until the tenth day of storage, while the control sample failed in TTA on the sixth day and the activated sample failed in pH on the eighth day, with the control sample failing on the fourth day of storage. However, in the case of overnight milk, the LPS-activated sample failed on the second day of storage, whereas the control sample failed on the first day of processing. Both LPS-activated and control samples failed on the first day of processing in TTA. The increase in shelf life when using LPS is less evident in overnight pasteurized milk, where the slight increase in the shelf life of the milk is not commercially significant

## 5. Conclusions

In this work, activation of the LPS can significantly reduce the growth of TBC, TCC, and *E. coli* in raw milk. In the present research, the treatment of raw milk using LPSs (prior to pasteurization) enhanced the shelf life of morning pasteurized milk by up to 10 days. The overall finding of the current study indicates that LPSs were successful in reducing bacterial growth in both raw and pasteurized milk under real-world conditions (dairy environment, milk production, handling, and transportation practices). However, LPS application alone is inadequate for enhancing the quality and accessibility of fresh milk from the dairy industry. Instead, a combination of hygienic practices and LPS application should be implemented, in order to have a better impact. We recommend LPS application should be majorly implemented at the milk collector level, to ensure the proper use of LPS chemical concentration and to prevent abuse on human health. Further studies should be conducted to evaluate the cost and benefit of implementing the LPS at the level of farmers, collectors, and processors.

## Figures and Tables

**Table 1:** Sample size and treatment of LPSs in each study area.

Study Site	Treatments	Farmers		Collectors		Factories		Total
		MM	OM	MM	OM	MM	OM	
Selale	Activated	10	10	5	5	1	1	32
	Control	10	10	5	5	1	1	32
Holeta	Activated	10	10	5	5	1	1	32
	Control	10	10	5	5	1	1	32
Asella	Activated	10	9	5	5	1	1	32
	Control	10	9	5	5	1	1	32
D/zeyit	Activated	9	9	5	5	1	1	30
	Control	9	9	5	5	1	1	30
Total		78	76	40	40	8	8	**250**

NB: MM = morning milk; OM = overnight milk (fresh evening milk spent over night).

**Table 2:** Effect of LPS activation on microbiological quality of raw milk along dairy value chain.

Value Chain	Bacteria	Sampling Time	N	Treatment	Mean	Std. Err.	*p*-Value
Farmers	Log10 TBC	Morning	39	Control	6.73	0.18	0.0003
			39	Activated	5.79	0.19	
		Overnight	38	Control	7.31	0.16	0.001
			38	Activated	6.55	0.17	
	Log10 TCC	Morning	39	Control	4.70	0.17	0.0002
			39	Activated	3.77	0.18	
		Overnight	38	Control	5.04	0.29	0.0196
			38	Activated	4.19	0.29	
	Log10 *E. coli*	Morning	39	Control	0.84	0.25	0.0067
			39	Activated	0.18	0.09	
		Overnight	38	Control	0.52	0.18	0.0022
			38	Activated	0.00	(omitted)	
Collectors	Log10 TBC	Morning	20	Control	6.99	0.12	1 × 10^−5^
			20	Activated	6.15	0.13	
		Overnight	20	Control	7.78	0.16	0.0008
			20	Activated	6.96	0.18	
	Log10 TCC	Morning	20	Control	5.63	0.11	1 × 10^−5^
			20	Activated	4.63	0.14	
		Overnight	20	Control	6.41	0.11	1 × 10^−5^
			20	Activated	5.27	0.14	
	Log10 *E. coli*	Morning	20	Control	0.93	0.31	0.0206
			20	Activated	0.20	0.16	
		Overnight	20	Control	0.63	0.25	0.0085
			20	Activated	0.00	(omitted)	

**Table 3:** Effect of LPSs on microbiological quality, as compared to Ethiopian standards.

Quality	Value Chain	Sampling Time	Treatment	N	N (% of Passes)	*p*-Value
TBC	Farmers	Morning	Activated	39	20 (51%)	0.04
			Control	39	11 (28%)	
		Overnight	Activated	38	15 (40%)	0.02
			Control	38	6 (16%)	
	Collectors	Morning	Activated	20	9 (45%)	0.001 *
			Control	20	-	
		Overnight	Activated	20	3 (15%)	0.072
			Control	20	-	
TCC	Farmers	Morning	Activated	39	32 (82%)	0.004 *
			Control	39	20 (51%)	
		Overnight	Activated	38	23 (60.5%)	0.039 *
			Control	38	14 (36.9%)	
	Collectors	Morning	Activated	20	14 (70%)	0.0001
			Control	20	1 (5%)	
		Overnight	Activated	20	6 (30%)	0.008
			Control	20	0	
*E-coli*	Farmers	Morning	Activated	39	35 (89.7%)	0.77
			Control	39	29 (74.4%)	
		Overnight	Activated	38	38 (100%)	0.005 *
			Control	38	31 (81.6%)	
	Collectors	Morning	Activated	20	18 (90%)	0.058
			Control	20	13 (65%)	
		Overnight	Activated	20	20 (100%)	0.017 *
			Control	20	15 (75%)	

Pass = the sum of very good and good quality, fail = very bad and bad quality, * indicates the presence of a significant difference between activated and control samples.

**Table 4:** Effect of LPS activation on mean pH, TTA, and thiocyanate of raw milk along dairy value chain.

Value Chain	Quality Test	Sampling Time	N	Treatment	Mean	Std. Err.	*p*-Value
Farmers	pH	Morning	39	Control	6.33	0.072	0.0359 *
			39	Activated	6.51	0.044	
		Overnight	38	Control	6.04	0.102	0.0288 *
			38	Activated	6.31	0.062	
	TTA (%)	Morning	39	Control	0.21	0.010	0.1087
			39	Activated	0.19	0.009	
		Overnight	38	Control	0.25	0.016	0.0019 *
			38	Activated	0.20	0.008	
	Thiocyanate (ppm)	Morning	39	Control	11.08	1.097	0.1238
			39	Activated	13.64	1.219	
		Overnight	38	Control	12.12	1.356	0.1565
			38	Activated	15.14	1.614	
Collectors	pH	Morning	20	Control	6.223	0.064	0.014
			20	Activated	6.404	0.046	
		Overnight	20	Control	6.056	0.061	0.0024
			20	Activated	6.304	0.055	
	TTA	Morning	20	Control	0.263	0.012	0.0143
			20	Activated	0.227	0.010	
		Overnight	20	Control	0.352	0.024	0.0054
			20	Activated	0.270	0.019	
	Thiocyanate (ppm)	Morning	20	Control	13.110	1.595	0.5155
			20	Activated	13.025	1.488	
		Overnight	20	Control	15.487	1.745	0.6731
			20	Activated	14.518	1.244	

* Indicates the presence of a significant difference (*p* < 0.05) between activated and control samples.

## Data Availability

The original contributions presented in the study are included in the article and Supplementary Materials, further inquiries can be directed to the corresponding author.

## Supplementary Materials

The following supporting information can be downloaded at: https://www.mdpi.com/article/10.3390/foods13081272/s1, Table S1: Raw data of the chemical and microbiological tests.
